# Supplementary material for: Chest pain in pediatric patients in the emergency department- Presentation, risk factors and outcomes-A systematic review and meta-analysis
Source: PLoS One. 2024 Apr 16;19(4):e0294461. doi: 10.1371/journal.pone.0294461 (PMC11020527; doi:10.1371/journal.pone.0294461)
Supplement: S1 Appendix — (DOCX) [file pone.0294461.s002.docx]

| **Source and search date** | **Search strategy** | **Results** |
| --- | --- | --- |
| **PubMed**  (NLM)  **Coverage:**  from database inception to search date  **Search date:**  2023-07-11 | ((((((((("Emergency Medicine"[Title/Abstract])) OR ("Emergency Service, Hospital"[Title/Abstract])) OR ("Evidence-Based Emergency Medicine"[Title/Abstract])) OR ("Pediatric Emergency Medicine"[Title/Abstract])) OR ("emergency room"[Title/Abstract])) OR ("emergency department"[Title/Abstract])) OR ("emergency patient"[Title/Abstract])) OR (ER[Title/Abstract])) OR (ED[Title/Abstract]) AND ((((((((((((("chest pain"[Title/Abstract]) OR (Pectoralgia[Title/Abstract])) OR (Stethalgia[Title/Abstract])) OR (Thoracalgia[Title/Abstract])) OR (Thoracodynia[Title/Abstract])) OR ("Heart Attack"[Title/Abstract])) OR (angina[Title/Abstract])) ) OR ("chest cramp"[Title/Abstract])) OR ("chest tightness"[Title/Abstract])) OR (heartburn[Title/Abstract])) | **6520** |
| **EMBASE**  (Elsevier, all sources included)  **Coverage:**  from database inception to search date  **Search date:**  2023-07-11 | ('emergency medicine':ab,ti OR 'emergency service, hospital':ab,ti OR 'evidence-based emergency medicine':ab,ti OR 'pediatric emergency medicine':ab,ti OR 'emergency room':ab,ti OR 'emergency department':ab,ti OR 'emergency patient':ab,ti OR er:ab,ti OR ed:ab,ti) AND ('chest pain':ab,ti OR pectoralgia:ab,ti OR stethalgia:ab,ti OR thoracalgia:ab,ti OR thoracodynia:ab,ti OR 'heart attack':ab,ti OR angina:ab,ti OR 'chest cramp':ab,ti OR 'chest tightness':ab,ti) | **13994** |
| **Web of Science**  (Core collection,  Clarivate)  **Coverage:**  from database inception to search date  **Search date:**  2023-07-11 | (TS=(("Emergency Medicine") OR ("Emergency Service, Hospital") OR ("Evidence-Based Emergency Medicine") OR ("Pediatric Emergency Medicine") OR ("emergency room") OR ("emergency department") OR ("emergency patient") OR (ER) OR (ED))) AND TS=((("chest pain") OR (Pectoralgia) OR (Stethalgia) OR (Thoracalgia) OR (Thoracodynia) OR ("Heart Attack") OR (angina) OR ("chest cramp") OR ("chest tightness"))) | **7913** |
| **Scopus**  (Elsevier)  **Coverage:**  from database inception to search date  **Search date:**  2023-07-11 | TITLE-ABS-KEY (("Emergency Medicine") OR ("Emergency Service, Hospital") OR ("Evidence-Based Emergency Medicine") OR ("Pediatric Emergency Medicine") OR ("emergency room") OR ("emergency department") OR ("emergency patient") OR (ER) OR (ED)) AND TITLE-ABS-KEY (("chest pain") OR (Pectoralgia) OR (Stethalgia) OR (Thoracalgia) OR (Thoracodynia) OR ("Heart Attack") OR (angina) OR ("chest cramp") OR ("chest tightness")) | **9233** |
| Total number of records identified | | **37660** |
| Total number of unique records after removing duplication | | **19891** |
