# Supplementary material for: Chest pain in pediatric patients in the emergency department- Presentation, risk factors and outcomes-A systematic review and meta-analysis
Source: PLoS One. 2024 Apr 16;19(4):e0294461. doi: 10.1371/journal.pone.0294461 (PMC11020527; doi:10.1371/journal.pone.0294461)
Supplement: S1 Table — (PDF) [file pone.0294461.s003.pdf]

Authors	Selection				Comparability of cohorts	Outcome			Total Score T
	**Representativeness of the exposed cohort**	**Selection of the non-exposed cohort**	**Ascertainment of exposure**	**Outcome of interest not present at study start**		**Assessment**	**Length of follow-up**	**Adequacy of follow-up of cohorts**	
Babbitt 2022	*	*	*		*	*			6
Brancato et al. 2021	*	*	*	*	*	*			6
Drossner et al. 2011	*	*	*	*	*	*			6
Gesuete et al. 2020	*	*	*	*	*	*	*	*	8
Hambrook et al. 2010	*	*	*	*	*	*			6
Lin et al. 2008	*	*	*	*	*	*			6
Massin et al. 2004	*	*	*	*	*	*	*	*	8
Mohan et al. 2018	*	*	*	*	*	*	*	*	8
Neff et al. 2012	*	*	*	*	*	*			6

Ocampo-Vázquez et al. 2019	*	*	*	*	*	*			6
Pissarra et al. 2022	*	*	*	*	*	*	*	*	8

Supplementary Table 1: Quality assessment of the studies. The Newcastle-Ottawa Quality Assessment Scale consists of 4 items on "study selection", 1 item on "comparability" and 3 items on "study outcome". According to this scale, studies can award one star for each of the 4 items on "selection" and for each of the 3 items on "outcome" and a maximum of 2 stars for "comparability". Higher number of stars indicates a higher quality study.
