# Supplementary material for: Chest pain in pediatric patients in the emergency department- Presentation, risk factors and outcomes-A systematic review and meta-analysis
Source: PLoS One. 2024 Apr 16;19(4):e0294461. doi: 10.1371/journal.pone.0294461 (PMC11020527; doi:10.1371/journal.pone.0294461)
Supplement: S2 Table — This table shows the certainty for the etiologies of chest pain. (PDF) [file pone.0294461.s004.pdf]

**Certainty Assessment**							**Effect**		
**Cardiac chest pain**									
**No. of Studies**	**Designs**	**Risk of Bias**	**Inconsistency (heterogeneity in MA)**	**Indirectness (External Validity)**	**Imprecision (Small SS, Wide CI)**	**Publication bias**	**pooled prevalence (95% CI)**	**Certainty**	**Importance**
**10**	Cohort	not serious	not serious	not serious	not serious	strongly suspected	(0.03, 95% CI [0.01, 0.04])	very low	Critical
**Certainty Assessment**							Effect		
**Non-cardiac chest pain**									
**No. of Studies**	**Designs**	**Risk of Bias**	**Inconsistency (heterogeneity in MA)**	**Indirectness (External Validity)**	**Imprecision (Small SS, Wide CI)**	**Publication bias**	**pooled prevalence (95% CI)**	Certainty	Importance
**10**	Cohort	not serious	not serious	not serious	not serious	strongly suspected	(0.98, 95% CI [0.96, 0.99])	very low	Important
**Certainty Assessment**							Effect		
**Respiratory chest pain**									
**No. of Studies**	**Designs**	**Risk of Bias**	**Inconsistency (heterogenei**	**Indirectness (External Validity)**	**Imprecision (Small SS, Wide CI)**	**Publication bias**	**pooled prevalence (95% CI)**	Certainty	Importance

			**ty in MA)**						
**8**	Cohort	not serious	not serious	not serious	not serious	strongly suspected	(0.13, 95% CI [0.09, 0.17])	very low	Important
**Certainty Assessment**							**Effect**	**Certainty**	**Importance**
**Gastrointestinal**									
**No. of Studies**	**Designs**	**Risk of Bias**	**Inconsistency (heterogeneity in MA)**	**Indirectness (External Validity)**	**Imprecision (Small SS, Wide CI)**	**Publication bias**	**pooled prevalence (95% CI)**		
**9**	Cohort	not serious	not serious	not serious	not serious	strongly suspected	(0.05, 95% CI [0.04, 0.07])	very low	Important
**Certainty Assessment**							**Effect**	**Certainty**	**Importance**
**Musculoskeletal**									
**No. of Studies**	**Designs**	**Risk of Bias**	**Inconsistency (heterogeneity in MA)**	**Indirectness (External Validity)**	**Imprecision (Small SS, Wide CI)**	**Publication bias**	**pooled prevalence (95% CI)**		
**9**	Cohort	not serious	serious	not serious	not serious	strongly suspected	(0.36, 95% CI [0.20, 0.51])	very low	Important

**Certainty Assessment**							**Effect**	**Certainty**	**Importance**
**Psychogenic**									
**No. of Studies**	Designs	Risk of Bias	Inconsistency (heterogeneity in MA)	Indirectness (External Validity)	Imprecision (Small SS, Wide CI)	Publication bias	**pooled prevalence (95% CI)**		
**6**	Cohort	not serious	serious	not serious	not serious	strongly suspected	(0.11, 95% CI [0.04, 0.18])	very low	Important

**Certainty Assessment**							**Effect**	**Certainty**	**Importance**
**Idiopathic**									
**No. of Studies**	**Designs**	**Risk of Bias**	**Inconsistency (heterogeneity in MA)**	**Indirectness (External Validity)**	**Imprecision (Small SS, Wide CI)**	**Publication bias**	**pooled prevalence (95% CI)**		
**4**	Cohort	not serious	serious	not serious	not serious	strongly suspected	(0.35, 95% CI [0.25, 0.45])	very low	Important
