# Supplementary material for: Chest pain in pediatric patients in the emergency department- Presentation, risk factors and outcomes-A systematic review and meta-analysis
Source: PLoS One. 2024 Apr 16;19(4):e0294461. doi: 10.1371/journal.pone.0294461 (PMC11020527; doi:10.1371/journal.pone.0294461)
Supplement: Attachment — Submitted filename: Review Comments.pdf [file pone.0294461.s005.pdf]

Review Comments

Journal Name: PLOS ONE
Title of the Manuscript: Presentation, Risk Factors, and Outcomes of Pediatric Patients who Present to the Emergency Department with Chest Pain a systematic review and meta-analysis
Manuscript Number: PONE-D-23-22358
Type of the Article: Research Article
Date of submission: September 22, 2023

For author;

1. The "**Title**" is inaccurate and incorrect. The "**Title**" modification is needed. Please, it is preferable to change it to "**Chest Pain in Pediatric Patients in the Emergency Department-Presentation, Risk Factors and Outcomes-A Systematic Review and Meta-analysis**".
2. Regarding the **initial phrase** of the "**Objective**" of the "**Abstract**"; (**This study sought to assess and determine**…) is inappropriate. Please, change it to '**This study aimed to assess and determine**…).
3. The author (s) mentioned in the "**Conclusion**" of the "**Abstract**" this sentence (**This review found musculoskeletal pain to the be most common cause of chest pain in children.**) then the author (s) in mentioned this sentence (**Review of our study population identified retrosternal chest pain to be the most common location of pain**.). Please, both **musculoskeletal pain** and **retrosternal chest pain** are relevant to the entity of "**location of pain**" no more. So, **both of the above sentences are contradictory and scientifically unacceptable**. Please, review both and edit them.
4. The "**Conclusion**" of the "**Abstract**" is scientifically very weak. Re-writing it again is needed.
5. In the "**Introduction**", for this sentence, "**Statistically, many children with chest pain have gastrointestinal etiologies with referred chest pain**", please, support your sentence with numbers and percentages.
6. In the "**Introduction**", also for this sentence, "**Statistically, many children with chest pain have gastrointestinal etiologies with referred chest pain**" is proceeded and followed with **general** writing sentences on "**chest pain**". This is not a good understanding flow. The author (s) should mention some sentences regarding general "**chest pain or chest pain in adults**" and then start to specify writing on the "**chest pain in pediatrics**" or use a comparative method between "**chest pain in children and adults**".
7. **Naming of investigators; even in abbreviations and their work** in this manuscript (**M.A., Z.N., J.P., M.S.Z** created the review protocol and a search strategy) in the "**Methods**" is a form of misplacement. So, please, transfer this sentence "I**nvestigators (M.A., Z.N., J.P., M.S.Z) created the review protocol and a search strategy.**" From "**Methods**" to the "**Author contribution statement**" section and change accordingly. Also, transfer this sentence "**Two assessors (Z.N. and J.P.) independently rated the quality of the studies using the Cochrane Collaboration's tool**". From "**Quality assessment of studies**" to the "**Author contribution statement**" section and change accordingly.
8. Please, where is the **reference for (Figure 1)** in the **text?!**
9. The following sentence in the "**Limitations**"; (**Second, many studies were observational and retrospective, which did not allow the authors to determine the precise cause of chest pain) is** incorrect and needs literature reviewing**.**

10. Regarding the final "**Conclusion**" it must be decisive, innovative, and expressive for the article. The "**Conclusion**" of the "**Abstract**" is also a brief reflective mirror of it.
11. Otherwise the included 2 studies **(Drossner 2011; reference No. 6) and Massin 2004; reference No. 9).** Where are the **final references and their citation** in the **tables** for the **remaining** included studies?!
12. There are several "**Grammatical**" mistakes. So, corrections and language editing are essential.

For Editor;

Dear sir,

Despite the article has sounding title, it is good in the field, I have no competing interests, and it is the only meta-analysis research regarding its title, there are defective editorial accuracy and innovative issues.

Thanks again for you.

Sincerely yours.

The reviewer

Dr. Yasser Mohammed Hassanain Elsayed

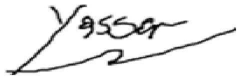

September 22, 2023

ORCID ID of the Reviewer; 0000-0003-1252-1615
Designation and Affiliation of the Reviewer; Scientist, independent researcher, critical care physician, and cardiologist at the Egyptian Ministry of Health
Submission Date*;* September 22, 2023
