# Supplementary material for: Chest pain in pediatric patients in the emergency department- Presentation, risk factors and outcomes-A systematic review and meta-analysis
Source: PLoS One. 2024 Apr 16;19(4):e0294461. doi: 10.1371/journal.pone.0294461 (PMC11020527; doi:10.1371/journal.pone.0294461)
Supplement: Attachment — Submitted filename: Response to Reviewers1.docx [file pone.0294461.s006.docx]

Dear esteemed reviewers,

Thank you so very much for returning our manuscript with your comments and suggestions. Please find our response to your comments below.

**Response to Journal Requirements:**

1. Thank you very much for your kindness in referencing the PLOS ONE style templates. We have reviewed them and adjusted the styling and format of the manuscript accordingly! Please let us know if there are still segments that are still not meeting the journal standards of formatting and style. Thank you!
2. Each figure was reviewed and ensured that a separate caption was given to each one.
3. We have pasted the tables as part of the main manuscript and removed the individual files. The supplementary tables have remained their own separate files.
4. The supplementary files were made into separate files and their legend was included at the end of the manuscript as per suggestion. Thank you!
5. This issue was resolved by removing the statement that referenced Table 3 as this was a mistake. We apologize for the confusion!
6. The caption and legends for the supplemental information was added to the end of the manuscript. Additionally we have updated some of the references accordingly with the journal style of formatting. Thank you for your guidance in this issue!
7. No additions or removals were made to the citation list.

Thank you so very much for the kind suggestions for the manuscript.

**Response to reviewers**

1. The title was changed as per the suggestion of the reviewer. Thank you!
2. This sentence was changed as per suggestions of the reviewer. Thank you!
3. We are having a little bit of difficulty understanding this point that was brought up. We understand musculoskeletal pain to be an etiology of pain as opposed to a means of localizing the pain. Retrosternal pain indicates a location in which any type of pain may be present. We are open to discussion and would love to further understand this issue to clarify it as much as possible for readers. Thank you for your help!
4. The conclusion of the abstract was re-written to be stronger. Thank you for pointing this out!
5. A reference was added to support this point. Thank you!
6. This portion of the introduction was changed as per suggestions of the reviewer. Thank you for advising us in making this section more coherent and better in flow!
7. The mentioned sentences were removed from their original locations and added to the author contributions portion of the manuscript. Thank you for clarifying this for us and making this wonderful suggestion!
8. The reference for Figure 1 was added into the text. Thank you!
9. This sentence was edited and the word observational was removed as there was some misunderstanding in this part. We meant to say only retrospective as this would make more sense because any physicians conducting the study would not be present at the time of patient encounter to directly examine the patients.
10. The conclusion was re-written to fall more in line with the reviewers suggestions. Thank you very much!
11. We have added in the references that were referred to in tables 1 and 2 that were used to conduct this study. Our apologies for this very obvious blunder. Thank you for your help in correcting this.
12. The manuscript was edited to correct for any grammatical errors.

We would like to thank the reviewers a tremendous amount for the time and dedication that they have put towards reviewing this manuscript and all other works that they are actively engaged in. Please let us know of any additional suggestions as we would love to further improve the manuscript and have it included in this prestigious journal. Thank you for your time!

Sincerely yours,

Mohammed Alsabri, MD,M.B.B.S, FAAP, FISQUA

ED Pediatrician, Pediatric Emergency Department, Woodhull Hospital, Brooklyn, NY, USA

EM and Critical care consultant Al-thawara Modern General Hospital

Confounder and Past President Of YAEMD

E-mail: [alsabri5000@gmail.com](mailto:alsabri5000@gmail.com)

Tel: 17186639248
