# Supplementary material for: Chest pain in pediatric patients in the emergency department- Presentation, risk factors and outcomes-A systematic review and meta-analysis
Source: PLoS One. 2024 Apr 16;19(4):e0294461. doi: 10.1371/journal.pone.0294461 (PMC11020527; doi:10.1371/journal.pone.0294461)
Supplement: Attachment — Submitted filename: Review Comments R1.docx [file pone.0294461.s007.docx]

**Review Comments R1**

**For author;**

Thank you to the authors for the positive response in the correction of many of the reviewer comments. But, there are still few comments.

**1.** Unfortunately, the “**Title; lines 1-3**” is still unchanged. I don’t know why?! The “**Title** and **abstract**” are the face of the article, interestingly, attractive for readers, Journals, Conferences selection for presentation, and also a mirror for the authors.

**2.** Regarding the following sentence in the “**Background**”, “**Statistically, many children with chest pain have gastrointestinal etiologies with referred chest pain**”.

Regrettably, the author changed it to “**However, the landscape shifts when considering pediatric cases, where statistical evidence points to gastrointestinal origins often leading to referred chest pain; lines 58-60**” with no response to the reviewer's request (**please, support your sentence with numbers and percentages**). I don’t know, why? **And there is no more difference** between the first and the modified sentence.

**3.** The author (s) added (**reference No. 14; line 60**) to the above sentence “**However, the landscape shifts when considering pediatric cases, where statistical evidence points to gastrointestinal origins often leading to referred chest pain [14]**” as the first reference in the text citation How?! **This is unacceptable**. Please, **arrange the references** in **serial order** (1, 2, 3, 4,5…etc.). **Serial order of the reference citing in the text is essential**.

4. Regarding the **addition of the defective references** that were referred to in (**Tables 1 and 2),** thank you. But why do you neglect to add **each No. of reference in brackets [] opposite each study ID in the tables rows** themselves. Please, add the No. of reference in brackets [] in the right place of the tables.

**5.** Regarding the “**Quality assessment of studies**”, the **reference** citing (**Kirmayr et al., 2021; lines 150**) is a missed in the final references. Please, add it to the final reference list and put it in the **text as a number** **in brackets** **[] with start this sentence with Kirmayr et al**.

**For the Editor.**

Dear sir,

I think that the correction of the above comments will improve the article's status.

Thanks again for you.

Sincerely yours.

***The reviewer***

**Dr. Yasser Mohammed Hassanain Elsayed**

*October 22, 2023*

Dr. Yasser Mohammed Hassanain Elsayed

**ORCID ID of the Reviewer**; [0000-0003-1252-1615](https://orcid.org/0000-0003-1252-1615)

Scientist, independent researcher, critical care physician, and cardiologist at the Egyptian Ministry of Health
